# Supplementary material for: An application of neighbourhoods in digraphs to the classification of binary dynamics
Source: Netw Neurosci. 2022 Jun 1;6(2):528–51. doi: 10.1162/netn_a_00228 (PMC9208003; doi:10.1162/netn_a_00228)
Supplement: Supplementary file 1 [file netn-06-528-s001.pdf]

Supplementary Materials for the Article

An application of neighbourhoods in digraphs to the classification of binary dynamics

Pedro Conceição[1], Dejan Govc[2], Jānis Lazovskis[3], Ran Levi[1], Henri Riihimäki[5] and Jason P. Smith[4]

[1] Institute of Mathematics, University of Aberdeen, Aberdeen, UK

[2] Faculty of Mathematics and Physics, University of Ljubljana, Ljubljana, Slovenia

[3] Riga Business School, Riga Technical University, Riga, Latvia

[4] Department of Mathematics and Physics, Nottingham Trent University, Nottingham, UK

[5] Department of Mathematics, KTH, Stockholm, Sweden

SUPPLEMENTARY MATERIAL

Counting directed 3-cliques

Let $\mathcal{G}$ be a digraph. For a vertex v_0 in $\mathcal{G}$ let I_{v_0}, O_{v_0} and R_{v_0} denote the number of in-, out- and reciprocal (both in and out) neighbours of v_0, respectively.

Lemma 1. *Let $\mathcal{G}$ be a digraph and let $v_0 \in \mathcal{G}$ be a vertex. Then the number of possible directed 3-cliques containing v_0 is given by*

$$\deg(v_0)(\deg(v_0) - 1) - (\mathrm{ind}(v_0)\mathrm{oud}(v_0) + R_{v_0}). \tag{1}$$

Proof. The set of in-neighbours of v_0 give rise to $2\binom{I_{v_0}}{2} = I_{v_0}(I_{v_0} - 1)$ directed 3-cliques containing v_0. Similarly the out-neighbours of v_0 give rise to $O_{v_0}(O_{v_0} - 1)$ directed 3-cliques containing v_0. A choice of each gives an extra $I_{v_0}O_{v_0}$ directed 3-cliques. Next, each reciprocal neighbour together with either an in-neighbour or an out-neighbour gives rise to three directed 3-cliques at v_0. The total number of those is $3R_{v_0}(I_{v_0} + O_{v_0})$. Finally, pairs of reciprocal neighbours give rise to six directed 3-cliques at v_0, and the total number of those is $6\binom{R_{v_0}}{2} = 3R_{v_0}(R_{v_0} - 1)$. Let $\mathsf{P}(v_0)$ denote the total number of transitive

Corresponding author: Ran Levi, r.levi@abdn.ac.uk

3-tournaments that can be formed by v_0 and its neighbours. Summing up we have

$$\begin{aligned}
\mathsf{P}(v_0) &= I_{v_0}(I_{v_0}-1) + O_{v_0}(O_{v_0}-1) + I_{v_0}O_{v_0} + 3R_{v_0}(I_{v_0}+O_{v_0}) + 3R_{v_0}(R_{v_0}-1) \\
&= (I_{v_0}-O_{v_0})^2 + 3(I_{v_0}O_{v_0} + R_{v_0}I_{v_0} + R_{v_0}O_{v_0} + R_{v_0}^2) - (3R_{v_0} + I_{v_0} + O_{v_0}) \\
&= (\mathrm{ind}(v_0) - \mathrm{oud}(v_0))^2 + 3\mathrm{ind}(v_0)\mathrm{oud}(v_0) - (\mathrm{ind}(v_0) + \mathrm{oud}(v_0)) - R_{v_0} \\
&= (\mathrm{ind}(v_0) + \mathrm{oud}(v_0))^2 - \mathrm{ind}(v_0)\mathrm{oud}(v_0) - \deg(v_0) - R_{v_0} \\
&= \deg(v_0)(\deg(v_0)-1) - (\mathrm{ind}(v_0)\mathrm{oud}(v_0) + R_{v_0})
\end{aligned}$$

as claimed. □

Size, distribution and structure of neighbourhoods in a sample digraph

We compare neighbourhoods in a sample digraph sorted by the parameters listed in Table S1 in terms of some structural features. The digraph $\mathcal{G}$ we use is the connectivity graph of the Blue Brain Project reconstruction of the cortical microcircuitry in a young rat brain Markram et al. (2015). The data we used is available at Project (2019). Our classification experiments are done on the same microcircuit. We also applied the same measurements to other collections of digraphs and obtained different results. Since our aim is primarily to examine possible relationship between structure and function, we do not report those results here. These extended results are presented at Aberdeen Neurotopology Group webpage.

We considered the top 50 vertices in the graph sorted by the parameters. For each parameter we computed the size in terms of number of vertices in each neighbourhood and the pairwise intersections, again in terms of the number of vertices in each intersection. In Table S1 we report the minimum, maximum and average of these numbers among the 50 neighbourhoods with highest value for each parameter. We also computed the first six Betti numbers of each neighbourhood and report the average of these numbers for each parameter. Finally, we considered the union of neighbourhoods in decreasing order, sorted by each parameter, and computed the number of neighbourhoods required to cover 90% of the neurons in the entire microcircuit (that is, 28,310 neurons).

We notice that the top 50 centres with respect to the last six parameters listed in Table S1 tend to generate neighbourhoods of size close or below the average, with relatively very small intersection. This correlates well with their capacity as selection parameters in our experiments (see Figure S3). However, the two

types of clustering coefficients, **fcc** and **tcc**, also generate small top neighbourhoods with small intersection, but are not exceptional as selection parameters.

We also examined the distribution of values for each parameter across the entire graph. The outcome is given in Figure S1, which visually justifies considering neighbourhoods with both highest and lowest parameter values. We did not find a correlation between the distribution of parameter values and their performance as selection or feature parameters.

We are therefore led to the conclusion that the performance of graph parameters as selection and/or feature parameters cannot be explained by the structural features we examined. This compares well with the conclusion drawn in Reimann et al. (2021), in which similar experiments using the same dataset but with a different methodology yield results that cannot be explained by structural features such as size and mutual intersection.

The coverage capability of neighbourhoods sorted by various graph and topological parameters is related to another graph theoretic concept. Let $\mathcal{G}$ be a digraph. If S is the entire vertex set of $\mathcal{G}$, then $N_{\mathcal{G}}(S) = \mathcal{G}$, but the converse is not true, as S may be much smaller than the full vertex set and still satisfy this condition. Subsets of vertices whose neighbourhoods are the entire graph are well studied in graph theory (Chartrand, Lesniak, & Zhang, 2016, Section 12.4).

Definition 1. *Let $\mathcal{G}$ be a finite digraph with vertex set V. A subset $S \subseteq V$ is a* dominating set *if $N_{\mathcal{G}}(S) = \mathcal{G}$. The minimum cardinality of a dominating set for $\mathcal{G}$ is called the* domination number *and is denoted by $\gamma(\mathcal{G})$. A dominating set of cardinality $\gamma(\mathcal{G})$ is said to be a* minimum dominating set.

Computing a minimal dominating set is known to be an NP hard problem Knuth (1974), though there exist good approximation algorithms. A good summary of the problem and common approaches appears in Li, Potru, and Shahrokhi (2020). In Table S1 we present, among other computations, the size of neighbourhoods and the number of neighbourhoods from a sorted list that it takes to cover 90% of the Blue Brain Project microcircuit. Depending on the selection parameter used, the results are quite different. This suggests that a choice of neighbourhoods informed by certain vertex parameters may give ways of producing more efficient approximation algorithms for the domination number of graphs.

Further Graph parameters

We describe here further graph and topological parameters we examined.

Degrees For each vertex v in a graph $\mathcal{G}$, its (total) *degree* $\deg(v)$ is the number of vertices in the open neighbourhood of v. The *in- and out-degree* of v, denoted $\mathrm{ind}(v)$ and $\mathrm{oud}(v)$ respectively, mean the number of in- and out-neighbours of v respectively. These invariants were examined as graph parameters in our classification algorithm and were found inefficient, except in the case of *size*, which is very closely related to degree and turns out to be the strongest feature parameter we found.

Reciprocal degree By the *reciprocal degree* of a vertex v we mean the number of neighbours that are both in-neighbours and out-neighbours. We used reciprocal degree in this work in two ways. The sum of all reciprocal degrees in a neighbourhood (abbreviated **rc**), and the reciprocal degree of the centre (**rc-centre**).

Density coefficients Every $(k+1)$-clique contains $k+1$ k-cliques. But no number of k-cliques in a graph is guaranteed to form any $(k+1)$-cliques. The *density coefficient* is a ratio of the number of $(k+1)$-cliques by that of k-cliques, normalised in its ambient graph.

Definition 2. *Let $\mathcal{G}$ be a digraph with n vertices. For $k \geq 2$ define the k-th* density coefficient *of $\mathcal{G}$ at v_0 by the formula*

$$D_k(v_0) \stackrel{\text{def}}{=} \frac{k}{(k+1)(n-k)} \cdot \frac{\mathsf{S}_k(v_0)}{\mathsf{S}_{k-1}(v_0)}.$$

The factor $k/(k+1)(n-k)$ normalises the invariant, so that $D_k(v_0) = 1$ for every $1 < k < n$ if v_0 is a vertex in $\mathcal{G}$ that is a complete digraph on n vertices. This is explained in the next lemma.

Lemma 2. *For each pair of natural numbers $0 < k < n$, any digraph $\mathcal{G}$ on n vertices, and any vertex v_0 in it,*

$$\frac{\mathsf{S}_k(v_0)}{\mathsf{S}_{k-1}(v_0)} \leq \frac{(k+1)(n-k)}{k}$$

with equality obtained if and only if $\mathcal{G}$ is a complete digraph on n vertices.

Proof. We prove the statement by a double counting argument closely following the one given in (Jukna, 2011, Section 10.4). Let U be the set of all pairs (τ, σ) where σ is a directed $(k+1)$-clique containing v_0

and $\tau \subseteq \sigma$ is a directed k-clique containing v_0. Then one can count the number of elements of U in two ways. First, the number of k-sub-cliques τ of a fixed $(k+1)$-clique σ containing v_0 is exactly k, therefore

$$|U| = k\mathsf{S}_k(v_0).$$

On the other hand, a fixed k-clique τ is a subclique of at most $(n-k)(k+1)$ distinct $(k+1)$-cliques σ, because there are $(n-k)$ different choices for a vertex that together with τ will form a $k+1$ clique, and once a vertex was chosen there are $(k+1)$ distinct orientations on the extra k edges, so that the outcome is a directed $(k+1)$-clique. Therefore,

$$|U| \leq (n-k)(k+1)\mathsf{S}_{k-1}(v_0).$$

Comparing the two expressions, we have:

$$k\mathsf{S}_k(v_0) \leq (n-k)(k+1)\mathsf{S}_{k-1}(v_0),$$

which, upon reordering gives the claimed upper bound. Computing the ratio for a complete digraph on n vertices shows that this upper bound is sharp. □

We remark that, while we use the density coefficients as vertex parameters, one can define a global density coefficient on a digraph $\mathcal{G}$ with vertex set V by

$$D_k(\mathcal{G}) \stackrel{\text{def}}{=} \frac{1}{|V|}\sum_{v\in V} D_k(v).$$

By Lemma 2, for any $2 \leq k \leq |V|-1$, $D_k(\mathcal{G}) = 1$ if and only if $\mathcal{G}$ is a complete digraph on V. Since any digraph on V is a subgraph of the complete digraph on V, $D_k(\mathcal{G})$ provides a set of numerical invariants for digraphs, parameterised by dimension (size of clique), which measure a notion of size of the digraph in comparison to the complete digraph on the same vertex set. In our specific application, density coefficients did not prove efficient as selection or feature parameters.

Digraph filtrations

Definition 3. *Let $\mathcal{G} = (V, E)$ be a digraph, and let Γ be a topological operator on digraphs. For a vertex $v \in V$, let $\Gamma_{\mathcal{G}}(v)$ denote $\Gamma(N_{\mathcal{G}}(v))$. If $S \subseteq V$ is any subset, let*

$$\Gamma_{\mathcal{G}}(S) \stackrel{\text{def}}{=} \Gamma(N_{\mathcal{G}}(S)) = \bigcup_{v\in S} \Gamma_{\mathcal{G}}(v).$$

Topological operators on digraphs respect inclusions, by definition, and therefore transform a digraph that is filtered by subgraphs into a space that is filtered by closed subspaces.

Definition 4. *Let $\mathcal{G} = (V, E)$ be a digraph and let Γ be a topological operator on digraphs. Fix a linear ordering $\omega\colon v_1 < v_2 < \cdots < v_M$ on V, where $|V| = M$. For any integer $n \geq 0$, let $S_n^\omega = \{v \in V \mid v \geq v_{M-n}\}$. Define a filtration $F_n^\omega(\Gamma(\mathcal{G})) \subseteq F_{n+1}^\omega(\Gamma(\mathcal{G})) \subseteq \cdots \subseteq \Gamma(\mathcal{G})$ by*

$$F_n^\omega(\Gamma(\mathcal{G})) \stackrel{\text{def}}{=} \Gamma_{\mathcal{G}}(S_n^\omega).$$

*The subspace $F_n^\omega(\Gamma(\mathcal{G}))$ will be referred to as the n-*th ω-filtration layer of $\Gamma(\mathcal{G})$.

From a data analysis point of view filtering $\Gamma(\mathcal{G})$, as proposed in Definition 4, can be applied in several ways. In particular, persistent homology Carlsson (2009) can be used to extract information from the topology in a way that is sensitive to the ordering chosen. As the orderings can be induced from various vertex functions, the filtrations enable probing into the effect these vertex functions have on the subspace topology. In other words, such filtrations give ways of building $\Gamma(\mathcal{G})$ as an increasing union of subspaces, and different choices of orderings may result in totally different sequences of subspaces. In this article we used graph and topological parameters to determine the ordering on vertices. We also considered only the top (or bottom) of the ordered lists of vertices, and hence studied only the bottom layers of the resulting filtrations.

Data and code

The data used is available at `https://doi.org/10.5281/zenodo.4290212`. The entire analysis code can be obtained from `https://github.com/JasonPSmith/TriDy`. The code for the NEST experiments is available at `https://github.com/jlazovskis/neurotop-nest/`. The computations for this paper were done using the Maxwell HPC cluster at the University of Aberdeen. To ensure the calculations were computed in a reasonable time frame we used a combination of parallelisation and publicly available packages with efficient algorithms. In particular, the structural parameters of each neighbourhood can be computed independently, so were done simultaneously across multiple nodes and cores. To compute many of the parameters standard Python packages were sufficient, such as numpy, scipy and networkx. However, for the more computationally intensive topological parameters we used variations of the Flagser software Lütgehetmann, Govc, Smith, and Levi (2020).

Supplementary Figures

**Parameter**	**size**			**intersection size**			**Betti numbers**						**90% cover**
	min	*max*	*avg*	*min*	*max*	*avg*	β_0	β_1	β_2	β_3	β_4	β_5	*centre count*
**fcc**	3	181	87.9	0	22	0.8	1	11	55	6	0	0	1591
**tcc**	3	170	86.2	0	22	0.6	1	10	49	5	0	0	1280
**ec**	1184	1633	1456.3	30	241	132.0	1	288	13237	2463	21	0	204
**nbc**	2	1184	589.9	0	132	21.6	1	142	3047	634	11	1	555
**size**	1417	1633	1509.7	44	241	130.3	1	287	11734	2310	19	0	179
**asg**	945	1604	1257.0	19	226	116.3	1	190	10362	3108	43	0	270
**asr**	1120	1622	1406.9	42	241	146.9	1	243	12603	3127	38	0	249
**blsg**	20	1344	555.2	0	96	12.9	1	111	1444	162	1	0	239
**blsr**	79	974	398.3	0	67	7.4	1	63	431	56	0	0	318
**clsg**	8	98	40.8	0	5	0.2	1	0	0	0	0	0	560
**clsr**	69	814	229.3	0	35	2.9	1	28	81	7	0	0	1297
**tpsg**	8	939	368.8	0	65	7.5	1	62	1077	131	1	0	445
**tpsr**	84	1166	524.4	0	98	11.3	1	101	1105	167	1	0	209
all vertices	2	1633	492.9	0	241	9.9	1	94	1032	146	1	0	212

Table S1. Size, pairwise intersections, average Betti numbers for the top 50 neighbourhoods of each parameter, and 90% coverage of the graph by neighbourhoods of highest valued centres, by each parameter. The last row is the same among all vertices, with the last entry on the right giving the average number required for 90% coverage over 50 random selections (without replacement) of centers.

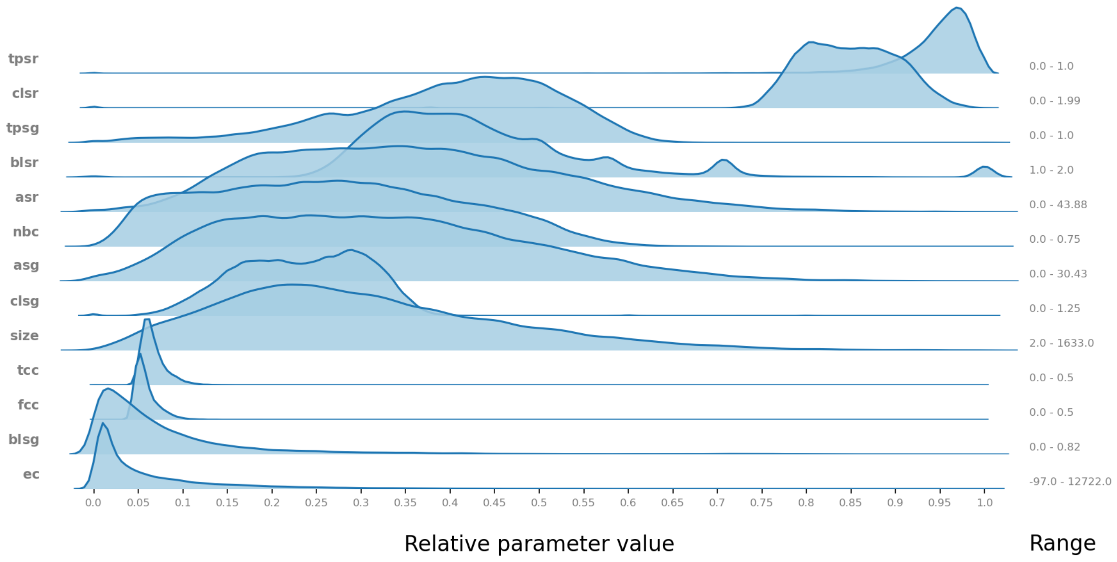

Figure S1. Distribution of parameter values across the entire Blue Brain Project microcircuit. The numbers on the right are minimum to maximum values. The values on the x-axis are the relative parameter values, rescaled from 0 to 1. Compare with Figure S4.

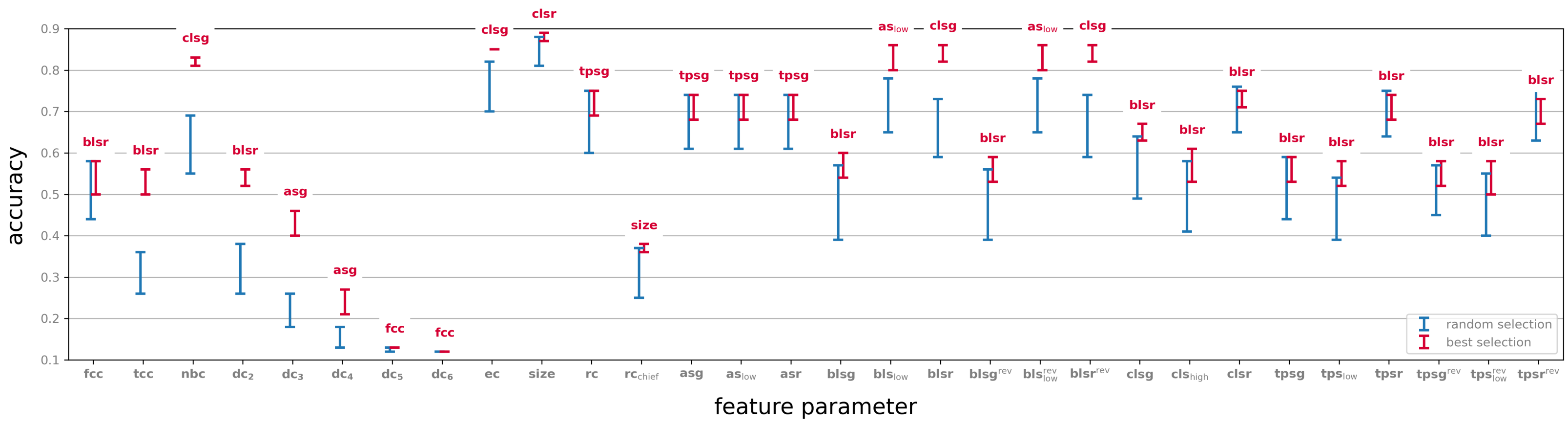

Figure S2. Classification accuracy of signals on the Blue Brain Project microcircuit using 50 randomly selected neighbourhoods, compared with accuracy of neighbourhoods selected by parameters with respect to the same feature parameter. Compare with Figure 5 in main article.

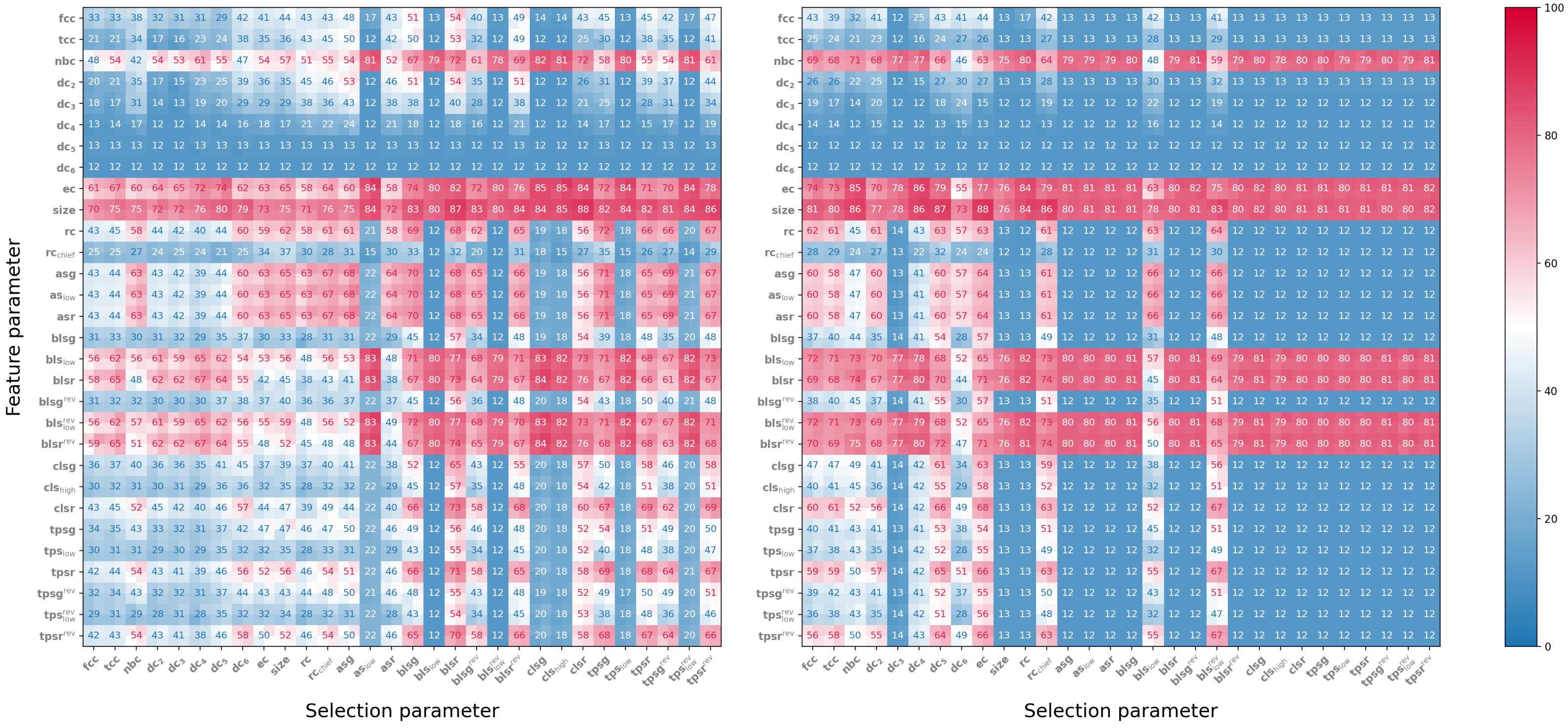

Figure S3. Results of classification experiments with respect to all parameters. Left: Classification accuracy with respect to 50 top value vertices by selection parameter. Right: Classification accuracy with respect to 50 bottom value vertices by selection parameter. Compare with Figure 4 in main article.

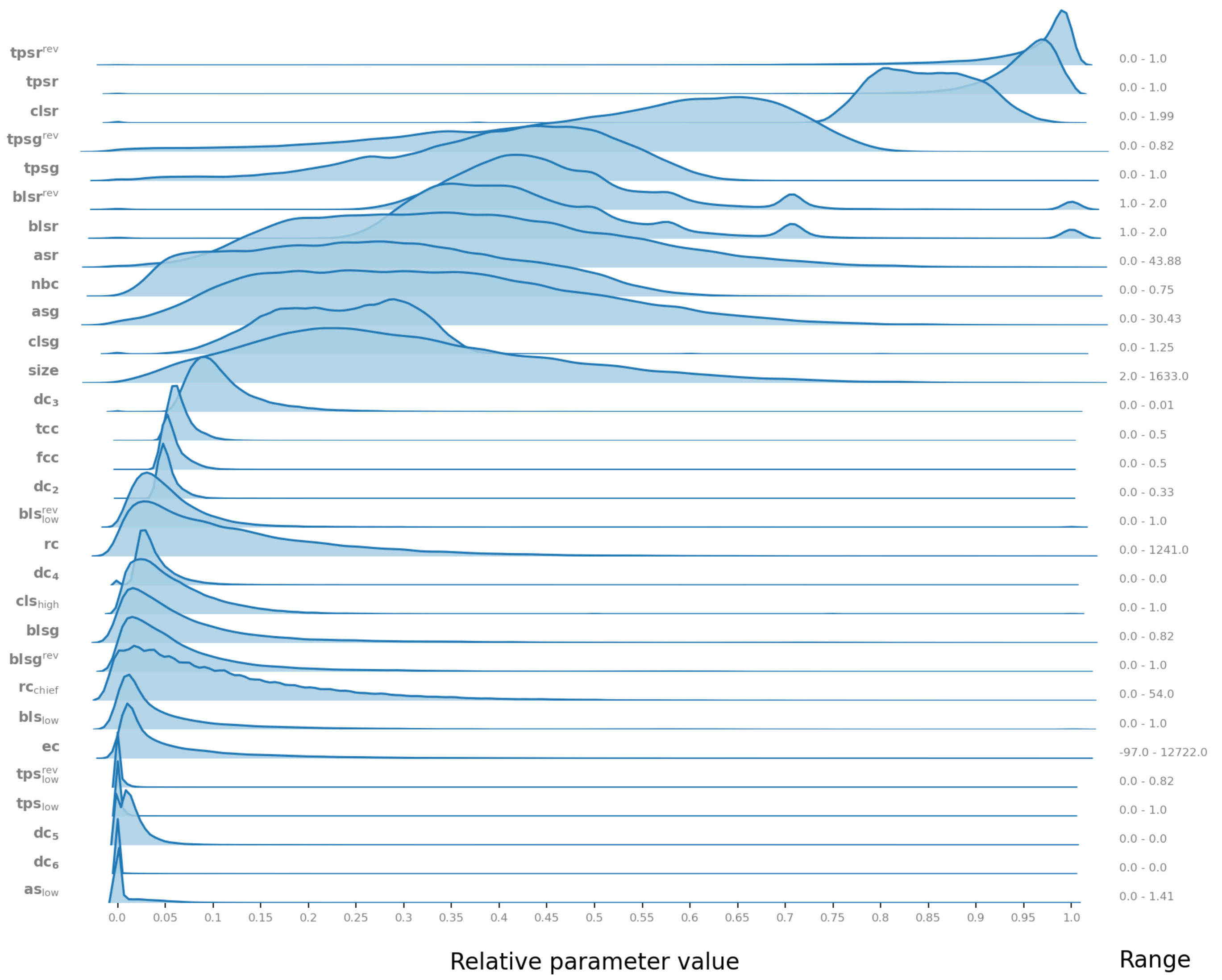

Figure S4. Distribution of all parameter values across the entire Blue Brain Project microcircuit. The numbers on the right are minimum to maximum values. The values on the x-axis are the relative parameter values, rescaled from 0 to 1. Compare with Figure S1.

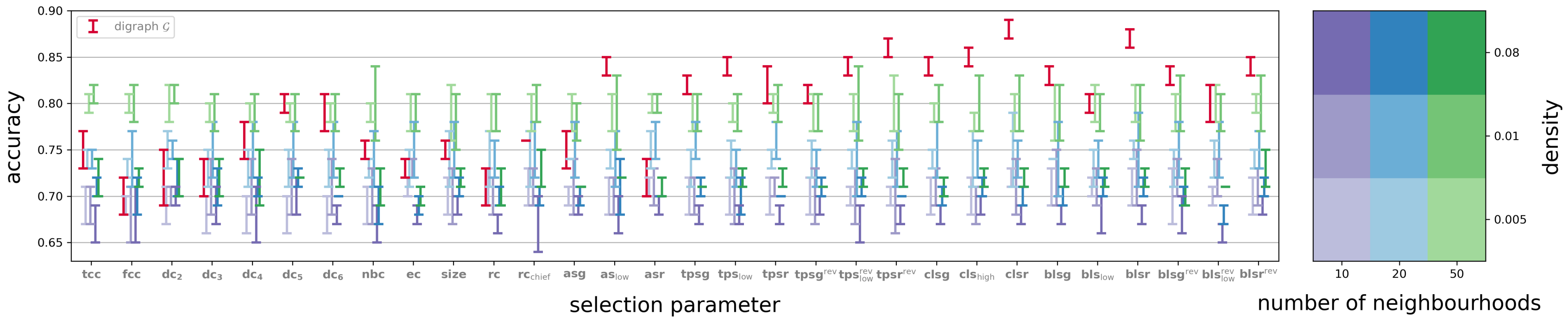

Figure S5. Classification of eight random signals on an Erdős–Rényi random digraph on 1000 vertices and connection probabilities of 8%, 1% and 0.5% and selection of 10, 20, and 50 neighbourhoods, modelled on a NEST simulator. Selection parameters from Figure 11 in the main article are included, along with additional parameters. Feature parameter is always **size**. Graph $\mathcal{G}$ means the Blue Brain Project graph and its performance with respect to **size** as feature parameter is given for comparison.

REFERENCES

Carlsson, G. (2009). Topology and data. *Bull. Amer. Math. Soc. (N.S.)*, *46*(2), 255–308. Retrieved from https://doi.org/10.1090/S0273-0979-09-01249-X doi: 10.1090/S0273-0979-09-01249-X

Chartrand, G., Lesniak, L., & Zhang, P. (2016). *Graphs and digraphs* (Sixth Edition ed.). CRC Press.

Jukna, S. (2011). *Extremal combinatorics* (Second edition ed.). Springer-Verlag.

Knuth, D. E. (1974, April). Postscript about np-hard problems. *SIGACT News*, *6*(2), 15–16. Retrieved from https://doi.org/10.1145/1008304.1008305 doi: 10.1145/1008304.1008305

Li, J., Potru, R., & Shahrokhi, F. (2020). A performance study of some approximation algorithms for computing a small dominating set in a graph. *Algorithms*, *13*(12). https://www.mdpi.com/1999-4893/13/12/339. doi: 10.3390/a13120339

Lütgehetmann, D., Govc, D., Smith, J. P., & Levi, R. (2020). Computing persistent homology of directed flag complexes. *Algorithms*, *13*(1). doi: 10.3390/a13010019

Markram, H., Muller, E., Srikanth Ramaswamy, M. W. R., ..., DeFelipe, J., Hill, S. L., ... Schürmann, F. (2015). Reconstruction and simulation of neocortical microcircuitry. *Cell*, *163*, 456-492.

Project, B. B. (2019). *Digital reconstruction of neocortical microcircuitry.* (Data retrieved from https://bbp.epfl.ch/nmc-portal/downloads)

Reimann, M., Riihimäki, H., Smith, J. P., Lazovskis, J., Pokorny, C., & Levi, R. (2021). Topology of synaptic connectivity constrains neuronal stimulus representation, predicting two complementary coding strategies. *BioArxiv*. https://doi.org/10.1101/2020.11.02.363929.
